# Atorvastatin Decreases Bone Loss, Inflammation and Oxidative Stress in Experimental Periodontitis

**DOI:** 10.1371/journal.pone.0075322

**Published:** 2013-10-10

**Authors:** Raimundo Fernandes de Araújo, Tatiana Oliveira Souza, Lígia Moreno de Moura, Kerginaldo Paulo Torres, Lélia Batista de Souza, Maria do Socorro Costa Feitosa Alves, Hugo Oliveira Rocha, Aurigena Antunes de Araújo

**Affiliations:** 1 Postgraduation Program in Functional and Structural Biology/Postgraduation Program Health Science/Department of Morphology, UFRN, Natal, RN, Brazil; 2 Postgraduation Program Health Science/UFRN, Natal, RN, Brazil; 3 Department of Dentistry/UNP, Postgraduation Program Public Health, UFRN, Natal, RN, Brazil; 4 Department of Biophysics and Pharmacology/UFRN, Natal, RN, Brazil; 5 Postgraduation Program Oral Pathology/UFRN, Natal, RN, Brazil; 6 Postgraduation Program Health Science/Postgraduation Program Public Health/UFRN, Natal, RN, Brazil; 7 Postgraduation Program in Biochemistry/UFRN, Natal, RN, Brazil; 8 Postgraduation Program Public Health/Postgraduation Program in Pharmaceutical Science/UFRN, Natal, RN, Brazil; Universidade Federal do Rio de Janeiro, Brazil

## Abstract

The aim of this study is to determine the effects of Atorvastatin treatment, an inhibitor of 3-hydroxy-3-methylglutaryl-coenzyme A (HMG-CoA) reductase, in periodontal disease. Male Wistar albino rats were randomly divided into five groups of ten rats each: (1) non-ligated treatment (NL), (2) ligature only (L), (3) ligature plus 1 mg/kg Atorvastatin daily for 10 days, (4) ligature plus 5 mg/kg Atorvastatin daily for 10 days, and (5) ligature plus 10 mg/kg Atorvastatin daily for 10 days. Following the treatment course, the periodontal tissue of the animals was analyzed by *Measurement of alveolar bone loss*, Histopathology and immunohistochemistry to determine of the expression of COX-2, MMP-2, MMP9, and RANKL/RANK/OPG. ELISA assay was used to quantitate the levels of IL-1β, IL-10, TNF-α, myeloperoxidase, malondialdehyde, and glutathione. The periodontal group treated with 10 mg/kg of Atorvastatin (3.9±0.9 mm; p<0.05) showed reverse the alveolar bone loss caused Experimental Periodontal Disease compared to (L) (7.02±0.17 mm). The periodontal group treated with 10 mg/kg of Atorvastatin showed a significant reduction in MPO and MDA (*p*<0.05) compared to ligature only group (L). Similarly in this group, the levels of the proinflammatory cytokines IL-1β and TNF-α were significantly decreased (*p*<0.05). Furthermore, MMP-2, MMP-9, RANKL/RANK, and COX-2 were all downregulated by Atorvastatin treatment, while OPG expression was increased. The findings support a role of Atorvastatin for reducing the bone loss, inflammatory response, oxidative stress, and expression of extracellular matrix proteins, while reducing RANK/RANKL and increase OPG in periodontal disease.

## Introduction

Atorvastatin is a member of the statin class of inhibitors. Through the inhibition of 3-hydroxy-3-methylglutaryl-coenzyme A (HMG-CoA) reductase, statins have revolutionized the treatment of hypercholesterolemia. The beneficial effects of HMG-CoA reductase inhibitors are usually attributed to their ability to reduce endogenous cholesterol synthesis. In addition to bleeding, statins are responsible for a variety of biochemical alterations, including a reduced accumulation of esterified cholesterol in macrophages, an increase in endothelial NO synthetase, a reduction of the inflammatory process, and an increased stability of atherosclerotic plaques [1].

Studies investigating Atorvastatin have shown that its treatment leads to significant reductions in the levels of proinflammatory cytokines (TNF, IL-1 and IL-6) [2]. In another study, Atorvastatin significantly decreased bone resorption markers, including levels of serum IL-6 [3]. Atorvastatin also decreased COX-2 expression within peripheral blood monocytes in patients with acute myocardial infarction [4] and increased IL-10 levels in a dose-dependent manner [5]. Atorvastatin has also been used to inhibit metalloproteinases [6], [7], osteoclastogenesis and bone destruction, and the expression of the receptor activator of nuclear factor-kappa B ligand (RANKL) [8].

It is yet unknown if Atorvastatin would be beneficial in a complex tissue such as the periodontium. In periodontal disease, increased inflammation and bone loss is modulated by the proinflammatory cytokines Il-1, Il-6, and TNF-α [9], [10]. These inflammatory cytokines further activate the expression of metalloproteinases and RANKL, ultimately contributing to periodontal bone loss [11]. The aim of the present study was to assess the effects of Atorvastatin in the treatment of periodontal disease by measuring levels of inflammation, antioxidants, matrix metalloproteinases, and bone markers after Atorvastatin treatment of rats with periodontal disease.

## Materials and Methods

### Animals

Experiments were performed on male Wistar rats (180–220 g) housed in standard conditions (12 h light/dark cycle and 22±0,1°C), with *ad libitum* access to standard diet (Presence/Evialis do Brasil Nutrição Animal LTDA, São Paulo) and water. The experimental protocol for experimental procedures and animal treatment was approved by the Animal Ethics Committee (number 28/2012) of the Federal University of Rio Grande Norte.

### Model for experimental periodontitis (EPD)

EPD was induced in rats under anaesthesia induced by ketamine (70 mg/kg administered i.p., 10% Quetamina, VETNIL, São Paulo) and xylazine (10 mg/kg adminstered i.p., 2% Calmium, São Paulo) by the placement of a sterile nylon thread ligature (3-0; Polysuture, NP45330, São Paulo) around the cervix of the maxillary left second molar. Eleven days after the initial treatment, the animals were euthanized with 20 mg/kg thiopental (0.5 g Thiopentax, Cristália, São Paulo).

### Drug treatments

For treatments, Atorvastatin (Lipitor 20mg Pfizer, São Paulo, Brazil) was solubilized in distilled water (vehicle). All treatments (Atorvastatin or vehicle) were given orally by gavage 1 h before ligation (induction of EPD) and thereafter once daily for 10 days. The animals were assigned randomly to the following five groups (10 animal for group): (1) a non-ligated group that received Water (NL), (2) a ligated group that received Water (L), (3) a ligated group treated with 1 mg/kg Atorvastatin (1 mg/kg Atorvast), (4) a ligated group a group treated with 5 mg/kg Atorvastatin (5 mg/kg Atorvast), and (5) a ligated group a group treated with 10 mg/kg Atorvastatin (10 mg/kg Atorvast).

### Measurement of alveolar bone loss (ABL)

The excised maxillae were fixed in 10% neutral formalin for 24 h. Both maxillary halves were then defleshed and stained with aqueous methylene blue (1%) to differentiate the bone from the tooth. Measurements of bone loss were made along the axis of each root surface of all molar teeth. Three recordings were made for the first molar teeth (three roots each) and two recordings for the second and third molar teeth (two roots earch).The total alveolar bone loss was obtained by taking the sum of the recordings from the buccal tooth surfaces and subtracting the values of the right maxilla(un ligated control) from the left maxilla, in millimetres [12]. Morphometric analysis of the alveolar bone was performed with standardized digital photography (OLYMPUS SC30), and the distance (millimeters) was measured with Image Software (analysis getIT 5.1). L group was used for baseline comparison.

### Histopathological analysis

The immunohistochemical analysis and the histological scores of the periodontal tissues were conducted by two calibrated oral pathologists. The sectioning was performed in the laboratoryof Morphology and Oral Pathology and subsequently analyzed by light microscopy in the Department of Morphology, UFRN. Alveolar bone specimens were harvested, fixed in 10% neutral-buffered formalin, and demineralized in 5% nitric acid. Following these treatments, the specimens were dehydrated, embedded in paraffin, and sectioned along the molars in a mesio-distal plane for haematoxylin-eosin staining. Sections of 4-µm thickness, corresponding to the area between the first and second molars where the ligature had been placed, were evaluated by light microscopy (40× magnification). Parameters such as inflammatory cell influx and the integrity of the alveolar bone and the cementum were analysed by a histologist in a single-blind fashion and graded as follows: A score of 0 indicates that inflammatory cell infiltration is absent or sparse and is restricted to the region of the marginal gingiva, and that the alveolar process and cementum are preserved; a score of 1 indicates moderate cellular infiltration (inflammatory cellular infiltration present on the entire gingival insert), minor alveolar process resorption, and an intact cementum; a score of 2 indicates accentuated cellular infiltration (inflammatory cellular infiltration present in the gingiva and in the periodontal ligament), accentuated degradation of the alveolar process, and partial destruction of the cementum; and a score of 3 indicates accentuated cellular infiltration, complete resorption of the alveolar process, and severe destruction of the cementum [13].

### Immunohistochemical analysis of COX-2, MMP-2, MMP-9, RANK-L, RANK and OPG

Thin sections of periodontal tissue (4 µm) were obtained with a microtome and were transferred to gelatine-coated slides. Each tissue section was then deparaffinised and rehydrated. The gingival and periodontal tissues slices were washed with 0.3% Triton X-100 in phosphate buffer, were quenched with endogenous peroxidase (3% hydrogen peroxide), and were incubated with the following primary antibodies (purchased from Santa Cruz Biotechnology) overnight at 4°C : cyclooxygenase-2 (COX-2), 1∶400; matrix metalloproteinase MMP-2, 1∶400; MMP-9, 1∶400; receptor activator of the NF-κB ligand (RANK-L), 1∶400; receptor activator of NF-κB (RANK), 1∶400; osteoprotegerin (OPG), 1∶400. After the slices were washed with phosphate buffer, they were incubated with a streptavidin-HRP-conjugated secondary antibody (Biocare Medical) for 30 minutes, and immunoreactivity to COX-2, MMP-2, MMP-9, RANK, RANK-L, and OPG was visualized using a colorimetric-based detection kit following the protocol provided by the manufacturer (TrekAvidin-HRP Label+Kit from Biocare Medical, DAKO, USA).

### Myeloperoxidase (MPO) assay

The relative level of neutrophil activity in the gingival samples was measured by assaying MPO. Gingival samples were harvested as described above and stored at −70°C until required for the assay. After homogenisation and centrifugation (2000× *g* for 20 min), MPO activity in these samples was determined by a colorimetric method described previously [14]. The results were reported as units of MPO per milligram of tissue.

### Malonaldehyde (MDA) levels

To assess lipid peroxidation, MDA production was measured with a thiobarbituric acid reaction in gingival tissue from the rats. Gingival tissue homogenate (0.25 ml of 10% tissue prepared in 0.15 M KCl) was added to a thiobarbituric acid solution (1.5 ml of 1% H_3_PO_4_ and 500 µl of a 0.6% thiobarbituric acid aqueous solution), and the mixture was placed in a water bath and heated for 45 min at 100°C. Next, 2 ml of n-butanol P.A. was added, and the mixture was homogenized and then centrifuged at 12,000 rpm for 15 min at 4°C. The absorbance of the butanol layer was measured at 520 nm (A1) and 535 nm (A2) (Genesys 10s UV-VIS, THERMA Scientific, England) The concentration of malonaldehyde was calculated as (A2 – A1), expressed as nmol of MDA per gram of gingival tissue.

### Glutathione (GSH) assay

GSH levels in gingival tissue were measured as a marker for antioxidant activity. The gingival samples were removed and stored at −70°C until required for the assay. Gingival tissue homogenate (0.25 ml of a 5% tissue solution prepared in 0.02 M EDTA) was added to 320 µl of distilled water and 80 µl of 50% TCA. The samples were then centrifuged at 3000 rpm for 15 min at 4°C. The supernatant (400 µl) was added to 800 µl of 0.4 M Tris buffer at pH 8.9 and 20 µl of 0.01 M DTNB. The absorbance of each sample was measured at 420 nm, and the results were reported as units of MPO per milligram of tissue.

### IL-1β, Il-10, and TNF-α assay

The gingival sample tissueswerestored at −70°C until required for each assay. The tissue collected was homogenized and processed as described by [15]. The levels of IL-1β, Il-10, and TNF-α in the gingival samples were determined with an ELISA commercial kit (R&D Systems, EUA) as described previously [16]. Briefly, micro titer plates were coated overnight at 4°C with antibodies against mouseTNF-α, IL-1β, and Il-10. After the plates were blocked, the samples and standards were added at various dilutions in duplicate and incubated at 4°C for 24 h. The plates were washed three times with buffer. The following antibodies were then added to the wells:biotinylated sheep polyclonal anti-TNF-α,anti-IL-1β, or anti-IL-10 (diluted 1∶1000 with 1% BSA assay buffer). After further incubation at room temperature for 1 h, the plates were washed, and 50 µl of avidin-HRP (diluted 1∶5000) was added. The color reagent o-phenylenediamine (OPD; 50 µl) was added 15 min later, and the plates were incubated in the dark at 37°C for 15–20 min. The enzyme reaction was stopped with H_2_SO_4_, and absorbance was measured at 490 nm. The resulting values were expressed in pg/ml.

### Statistical analysis

The data are presented as means+standard error of the mean (SEM) or as medians, where appropriate. Analysis of Variance (ANOVA) followed by Bonferroni's test was used to calculate the means, and the Kruskal–Wallis test followed by Dunn's test was used to compare medians (GraphPad PRISM 5.0 Software). A *P*-value of <0.05 was considered to indicate a significant difference.

## Results

### Effect of Atorvastatin treatment on alveolar bone loss in rats with EPD

**Figure 1:**
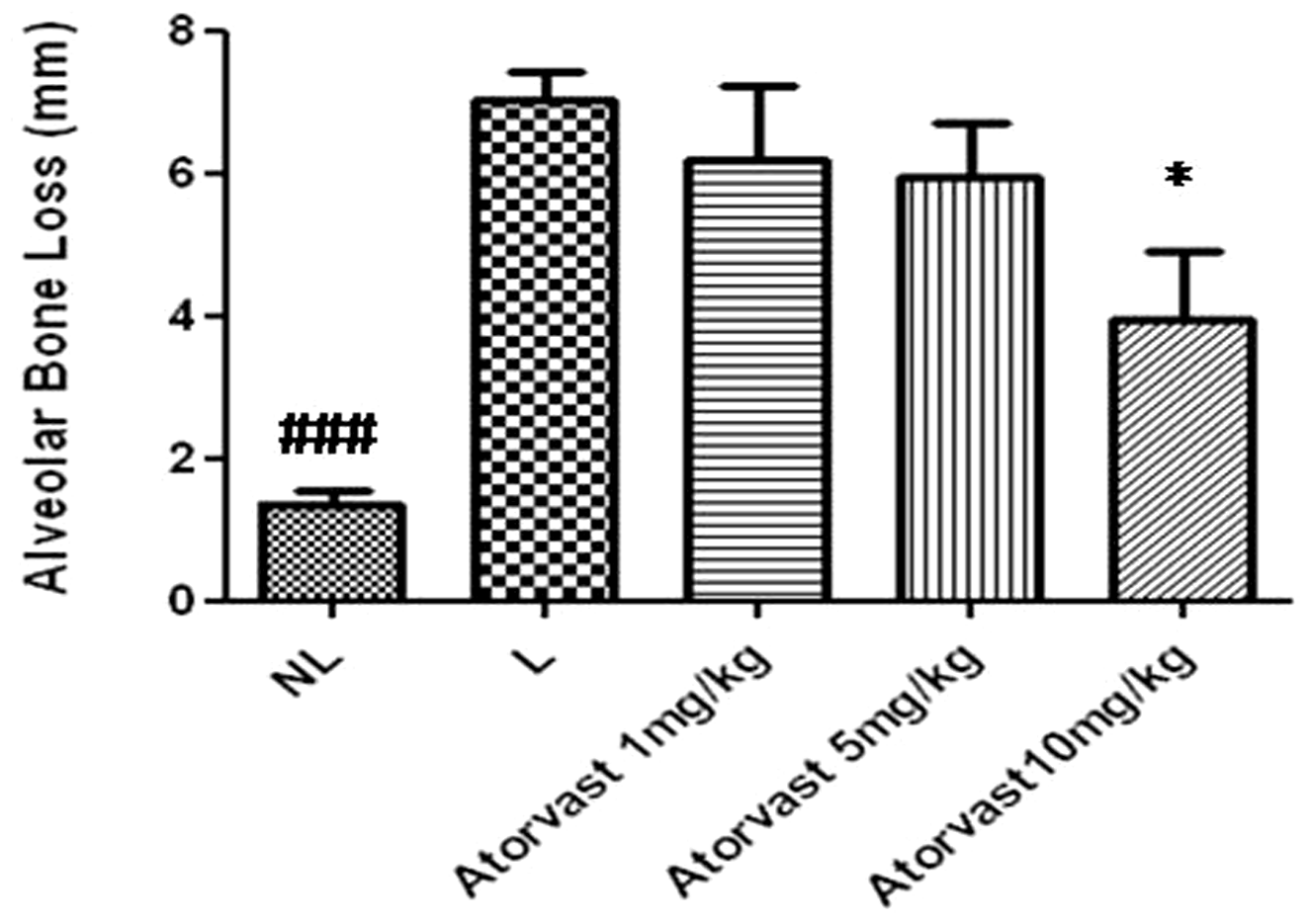
Effect of Atorvastatin treatment on alveolar bone loss associated with experimental periodontitis Disease (EPD) in rats. Values are expressed as means± SEM (###p<0.001, *p<0.05; determined with ANOVA and Tukey'stest).

**Figure 2:**
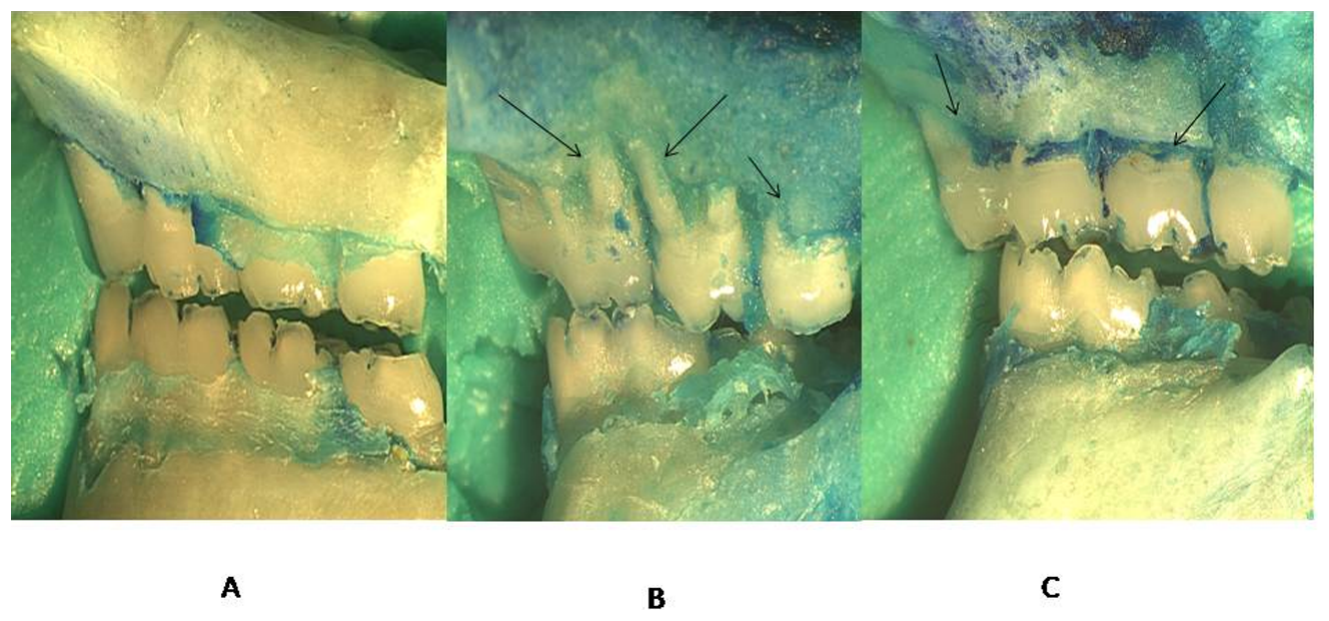
Clinical characteristics of teeth and periodontal tissue. (A) Samples from the NL group, with no resorption of the alveolar bone. (B) Samples from the L group, where severe bone resorption with root exposure is observed (Arrows). (C) Samples from the L group treated with Atorvastatin 10 mg/Kg, where decreased bone resorption is observed (Arrows). Images were obtained at an original magnification of 1.7×.

Rats with EPD (L) showed a significant alveolar bone loss compared to NL (NL = 1.4±0.07 mm; L = 7.02±0.17 mm; p<0.001). It was observed that treatment with Atorvastatin 10 mg/Kg reverse the alveolar bone loss caused by EPD (Atorv 10 mg/Kg 3.9±0.9; p<0.05) (Fig. 1). These data are shown in Fig. 2.A, which shows the macroscopic aspects of NL group (SO) with no resorption of the alveolar bone when compared to the L group (EPD), where severe bone resorption with root exposure is observed (Fig. 2B). Fig. 2C shows the macroscopic appearance of periodontium subjected to EPD and treated with Atorvastatin 10 mg/kg, where decreased bone loss is observed. Rats treated with low levels of Atorvastatin (1 mg/kg and 5 mg/kg) had no significant differences inalveolar bone loss compared to the L group (L = 7.02±0.17 mm; Atorv 1 mg/kg = 6.9±0.1 mm; Atorv 5 mg/kg = 5.8±0.6 mm;*p*>0.05), as seen in Figure 1.

### Histopathological analysis

**Figure 3:**
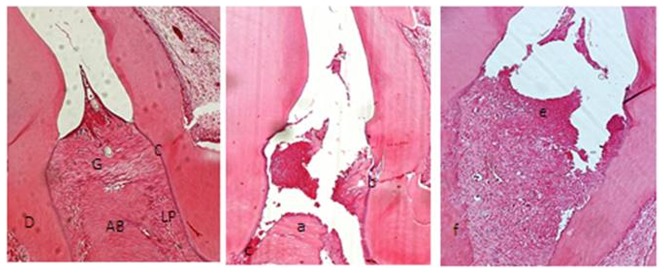
Microscopic analysis. (A) Normal periodontium and (B) periodontium from a rat presenting with periodontitis (treated with saline) showing alveolar bone and cementumresorption and inflammatory cell infiltration. (C) Reduced inflammation and alveolar bone loss in the periodontium of rats treated with Atorvastatin (10 mg/kg) for 10 days. Sections were stained with H&E. Microscopic original magnification at 40×. Scale bars = 100 µm. G = gingiva; PL = Periodontal ligament; D = dentin; AB = alveolar bone; C = Cementum; a = increased bone loss in AB; b = resorption of cementum; c = inflammatory process in PL and intense destruction of collagen fibers in the PL; e = decreased inflammation process in PL anddiscrete destruction ofcollagen fibers in PL; f = decreasedbone loss in AB.

Rats with periodontal disease that were treated with a high dose of Atorvastatin (10 mg/kg) had significantly less alveolar bone loss then similar animals that were treated with a low dose of Atorvastatin (1 mg/kg; *p*<0.05). As seen in Figure 3A, we detected a discrete cellular infiltration (restricted within the gingival margin), preserved alveolar process, and cementum in the rats with periodontitis that were treated with high-dose Atorvastatin for 10 days. This group also had a reduced level of inflammation and alveolar bone loss in their periodontium.

A histological analysis of the region between the first and second molars of control animals was representative of the structure of a normal periodontium, in that the gingiva, periodontal ligament, alveolar bone, and cementum can all be observed (Figure 3). Conversely, the histopathology of the periodontium of untreated animals subjected to experimental periodontitis (L) revealed inflammatory cell infiltration coupled with severe cementum and alveolar process destruction (Figure 3B; Table 1) and received a median score of 3. Animals with periodontitis that were treated with a high dose of Atorvastatin showed reduced inflammatory parameters (Figure 3C) and had a median score of 2 (range: 1–2; Table 1).

**Table 1:** Histological analysis of maxillae from rats presenting with periodontal disease, Natal, RN, 2013.

Normal	PD	ATOVAST1 mg/Kg	ATOVAST5 mg/Kg	ATOVAST10 mg/Kg
0 (0-0)	3 (3-3)*	3 (3-3)*	2 (2-3)	2(1-2)

* p<0.05.

### Immunohistochemical detection of COX-2, MMP-2, MMP-9, RANK-L, RANK, and OPG

**Figure 4:**
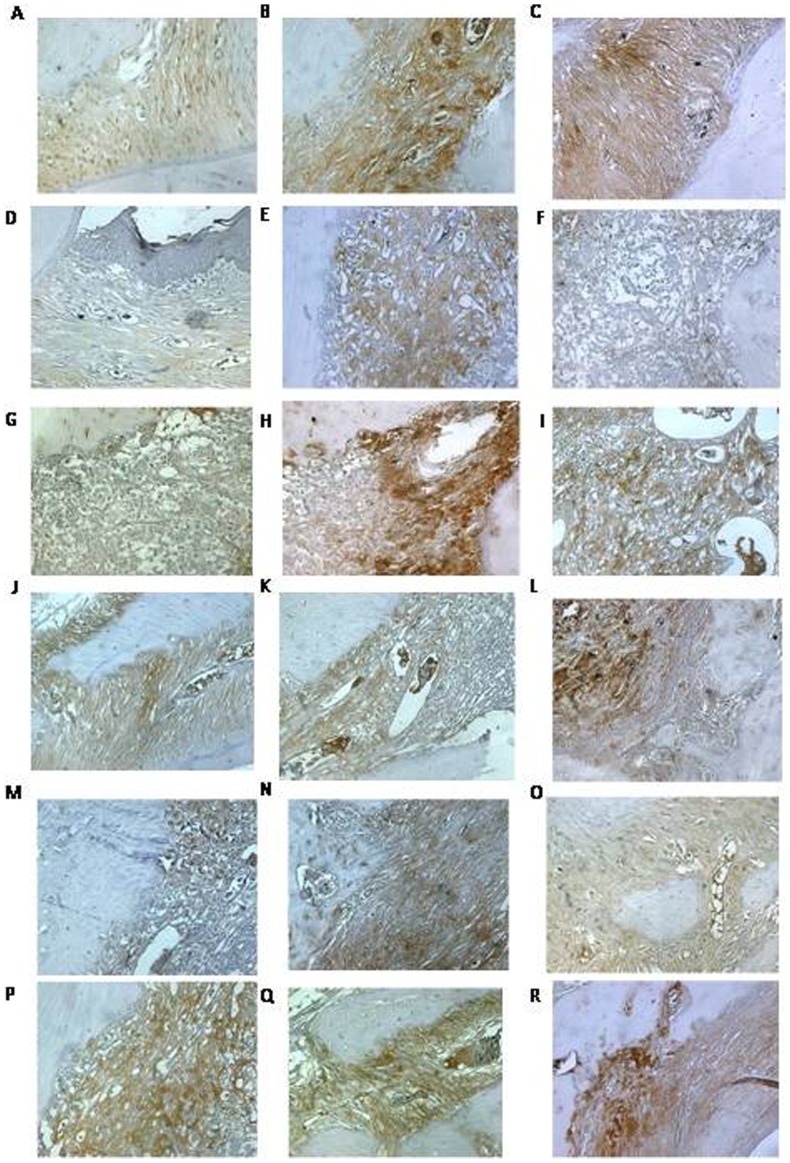
Histological analyses of (A–C) MMP-2, (D-F) MMP-9, (G–I) COX-2, (J–L) RANK, (M–O) RANK-L, and (P–R) OPG in periodontal tissue of rats with periodontal disease. Rats subjected to saline are pictured in A, D, G, J, M, P; rats with periodontal disease are pictured in B, E, H, K, N, Q; rats with periodontal disease and treated with Atorvastatin (10 mg/kg) are pictured in C, F, I, L, O, R. 100× magnification, bar scale = 100 µm.

The periodontium of untreated rats with experimental periodontitis showed marked immune-staining for COX-2, MMP-2, MMP-9, RANK-L, and RANK (Figures 4B, 4E, 4H, 4K, and 4N), compared to normal rats treated with saline alone (Figures 4A, 4D, 4G, 4J, and 4M). Furthermore, the periodontium of rats with periodontitis and treated with a high-dose of Atorvastatin had even lower levels of COX-2, as well as MMP-2, MMP-9, RANK-L and RANK (Figures 4C, 4F, 4I, 4L, and 4O). In comparison to control animals, the staining for osteoprotegerin (OPG) was moderately increased in the with periodontitis (L) and significantly increased in the animals with periodontitis that were treated with a high dose of Atorvastatin.

### Reduction of the inflammatory response

**Figure 5:**
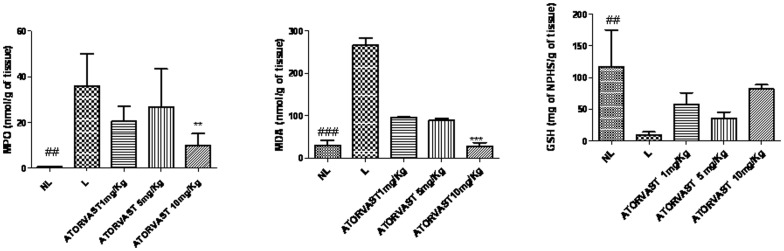
Levels of myeloperoxidase (MPO), malondialdehyde (MDA), and glutathione (GSH) in control animals with no ligature (NL), animals with periodontal disease induced by a ligature and treated with saline (L), or animals with periodontal disease and treated with 1, 5, or 10 mg/kg of Atorvastatin (#; * *p*<0.05;).

**Figure 6:**
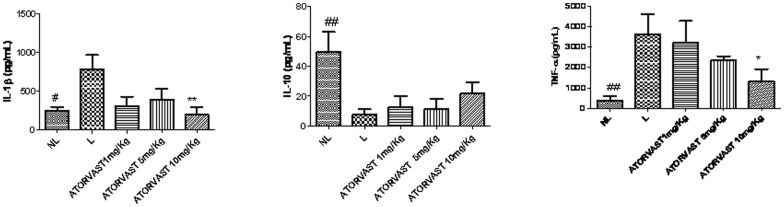
Levels of IL-1β, Il-10, and TNF-α in normal control animals with no ligature (NL), animals with periodontal disease induced by a ligature and treated with saline (L), or animals with periodontal disease and treated with 1, 5, or 10 mg/kg of Atorvastatin (#; *p<0.05; **p<0.01; ***p<0.001).

The myeloperoxidase (MPO) activity in each group with periodontal disease (L) was significantly increased in comparison with the control group (NL; *p*<0.01). The group with periodontitis that was treated with the high dose of Atorvastatin (10 mg/kg) showed a significant reduction in the concentration of MPO (*p*<0.01; Figure 5). The levels of the proinflammatory cytokines IL-1β and TNF-α were also significantly decreased in this group (*p*<0.05; Figure 6). The group treated with 1 mg/kg and 5 mg/kg Atorvastatin had the same levels of MPO, IL-1β, and TNF-α compared to the Lgroup (*p*>0.05) (Figure 5).

### Reduction of oxidative stress

Compared to saline, treatment with a high dose of Atorvastatin in animals with periodontal disease significantly reduced malondialdehyde (MDA) activity (*p*<0.001), treatment with 1 mg/kg and 5 mg/kg Atorvastatin reduced the activity of MDA (*p*<0.05), while glutathione (GSH) levels were increased, although not statistically different (Figure 5).

## Discussion

Indeed, the study that was utilized in this study is an animal model where periodontitis is induced by the intraoral placement of a nylon thread,making it a trauma-based periodontal disease model [17]. The initial immune response in chronic periodontitis occurs following colonization of the gingival sulcus by periodontopathic bacteria. The presence of the bacteria induces the production of cytokines and chemokines by the gingival epithelium. This results in the expression of adhesion molecules, increased permeability of gingival capillaries and chemotaxis of polymorphonuclear neutrophils through the junctional epithelium and into the gingival sulcus. The specific cytokines and chemokines produced by this initial response lead to a perivascular T-cell/macrophage dominated inflammatory infiltrate in the connective tissues. [18]. The primary mediators of periodontal inflammation are prostaglandins (PG; mainly PGE2) and the cytokines interleukin-1 (IL-1) and tumour necrosis factor alpha (TNF-α). [19].

A study using an animal model of oral mucositis demonstrated that Atorvastatin significantly reduced TNF-α and IL-1β levels [20]. These same findings were observed in a model of rheumatoid arthritis [21]. In this study, using a model of periodontal disease, animals treated with 10 mg/kg of Atorvastatin also had reduced levels of TNF-α and IL-1β. Atorvastatin's anti-inflammatory activity is evidenced by the reduced expression of COX-2 (an important enzyme selectively induced in inflamed tissue) in the periodontal tissue. The observed reduction in myeloperoxidase levels further confirms the reduction of leukocyte migration in treated animals. The outcomes related to the activity of Atorvastatin suggest that periodontal disease development involves an intricate signalling pathway that encompasses metalloproteinase expression and proteins linked to bone activity. Animals treated with 1 mg/kg and 5 mg/kg of Atorvastatin did not show reduced bone loss, anti-inflammatory, and/or antioxidant activity, possibly because the effects of Atorvastatin treatment are dose dependant and only observed at 10 mg/kg.

Matrix metalloproteinases (MMPs) are zinc- and calcium-dependent endopeptidases that function at a neutral pH. Fibrillar collagens are the major components of periodontal extracellular matrix and, during pathologic conditions, these collagens are further cleaved by active gelatinases (MMP-2 and MMP-9) [22]. Our results demonstrate that a 10-day treatment of Atorvastatin inhibited MMP-2 and MMP-9. This observation is very important since both MMP-2 and MMP-9 are strongly expressed during the acute phase of periodontitis (7–21 days) [23].

Another activity observed in the evolution of periodontal disease is the bone remodelling process dependent on a balance between the RANKL and OPG [24]. RANKL, a member of the TNFR family, exists in both a soluble and a membrane-bound form and its mRNA is expressed by osteoblastic lineage cells. RANKL stimulates osteoclasts to differentiate and inhibits their apoptosis. Furthermore, it binds to osteoclasts [25]. OPG, on the other hand, is a physiological humoral regulator of osteoclast-mediated bone resorption. OPG appears to function as a secreted tumour necrosis factor receptor (TNFR)-related protein and as a regulator of bone density, which can act locally and systemically by down-regulating osteoclast maturation [26].

In the present study, the reduction of the expression of RANKL in tissue after treatment with Atorvastatin, coupled with the strong staining of OPG, in tissue. Host-modulatory therapies for periodontitis, including the use of non-steroidal anti-inflammatory drugs (NSAIDs), have been shown to prevent formation of prostaglandins, prostacyclin, and thromboxane, due to a blockage of cyclooxygenase (COX) enzyme. In periodontal diseases, prostaglandin E_2_ has been extensively correlated with inflammation and bone resorption [27]. The side effects of NSAIDs, mainly cardiovascular, limit its use [28]. Another two classes of drugs, angiotensin II type 1 receptor (AT1) blocker [29] and β-blockers [30], have been investigated for periodontal disease and also found to reduce cytokine levels. These studies corroborate the results found by our study pertaining to Atorvastatin. In the future, there is a need to investigate the signaling pathways that are affected to better understand how can interfere with bone loss.

In conclusion, in periodontal disease, we find that Atorvastatin has anti-inflammatory activity, as indicated by reduced cytokine expression, and stress oxidative. These findings were further supported by a reduced expression of MMP-2 and MMP-9 in tissue, coupled with an increase in OPG and a decrease in RANKL -.
